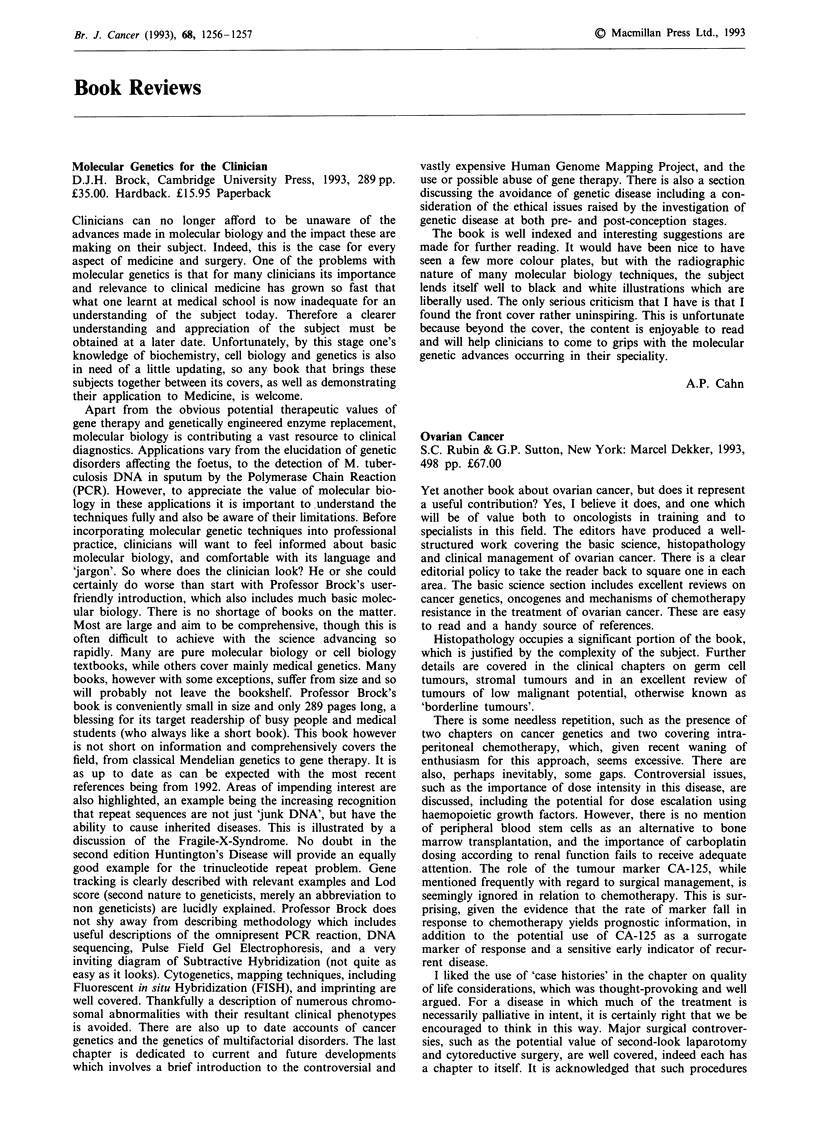# Molecular Genetics for the Clinician

**Published:** 1993-12

**Authors:** A.P. Cahn


 © Macmillan Press Ltd., 1993

### Book Reviews

### Molecular Genetics for the Clinician

D.J.H. Brock, Cambridge University Press, 1993, 289 pp. £35.00. Hardback. £15.95 Paperback

Clinicians can no longer afford to be unaware of the advances made in molecular biology and the impact these are making on their subject. Indeed, this is the case for every aspect of medicine and surgery. One of the problems with molecular genetics is that for many clinicians its importance and relevance to clinical medicine has grown so fast that what one learnt at medical school is now inadequate for an understanding of the subject today. Therefore a clearer understanding and appreciation of the subject must be obtained at a later date. Unfortunately, by this stage one's knowledge of biochemistry, cell biology and genetics is also in need of a little updating, so any book that brings these subjects together between its covers, as well as demonstrating their application to Medicine, is welcome.

Apart from the obvious potential therapeutic values of gene therapy and genetically engineered enzyme replacement, molecular biology is contributing a vast resource to clinical diagnostics. Applications vary from the elucidation of genetic disorders affecting the foetus, to the detection of M. tuberculosis DNA in sputum by the Polymerase Chain Reaction (PCR). However, to appreciate the value of molecular biology in these applications it is important to understand the techniques fully and also be aware of their limitations. Before incorporating molecular genetic techniques into professional practice, clinicians will want to feel informed about basic molecular biology, and comfortable with its language and 'jargon'. So where does the clinician look? He or she could certainly do worse than start with Professor Brock's user-friendly introduction, which also includes much basic molecular biology. There is no shortage of books on the matter. Most are large and aim to be comprehensive, though this is often difficult to achieve with the science advancing so rapidly. Many are pure molecular biology or cell biology textbooks, while others cover mainly medical genetics. Many books, however with some exceptions, suffer from size and so will probably not leave the bookshelf. Professor Brock's book is conveniently small in size and only 289 pages long, a blessing for its target readership of busy people and medical students (who always like a short book). This book however is not short on information and comprehensively covers the field, from classical Mendelian genetics to gene therapy. It is as up to date as can be expected with the most recent references being from 1992. Areas of impending interest are also highlighted, an example being the increasing recognition that repeat sequences are not just 'junk DNA', but have the ability to cause inherited diseases. This is illustrated by a discussion of the Fragile-X-Syndrome. No doubt in the second edition Huntington's Disease will provide an equally good example for the trinucleotide repeat problem. Gene tracking is clearly described with relevant examples and Lod score (second nature to geneticists, merely an abbreviation to non geneticists) are lucidly explained. Professor Brock does not shy away from describing methodology which includes useful descriptions of the omnipresent PCR reaction, DNA sequencing, Pulse Field Gel Electrophoresis, and a very inviting diagram of Subtractive Hybridization (not quite as easy as it looks). Cytogenetics, mapping techniques, including Fluorescent *in situ* Hybridization (FISH), and imprinting are well covered. Thankfully a description of numerous chromosomal abnormalities with their resultant clinical phenotypes is avoided. There are also up to date accounts of cancer genetics and the genetics of multifactorial disorders. The last chapter is dedicated to current and future developments which involves a brief introduction to the controversial and vastly expensive Human Genome Mapping Project, and the use or possible abuse of gene therapy. There is also a section discussing the avoidance of genetic disease including a consideration of the ethical issues raised by the investigation of genetic disease at both pre- and post-conception stages.

The book is well indexed and interesting suggestions are made for further reading. It would have been nice to have seen a few more colour plates, but with the radiographic nature of many molecular biology techniques, the subject lends itself well to black and white illustrations which are liberally used. The only serious criticism that I have is that I found the front cover rather uninspiring. This is unfortunate because beyond the cover, the content is enjoyable to read and will help clinicians to come to grips with the molecular genetic advances occurring in their speciality.

A.P. Cahn